# Comparison of Updated Weight and Height Percentiles with Previous References in 6-17-Year-Old Children in Kayseri, Turkey

**DOI:** 10.4274/jcrpe.3482

**Published:** 2017-03-01

**Authors:** Gökmen Zararsız, Betül Çiçek, Meda Kondolot, M. Mümtaz Mazıcıoğlu, Ahmet Öztürk, Selim Kurtoğlu

**Affiliations:** 1 Erciyes University Faculty of Medicine, Department of Biostatistics, Kayseri, Turkey; 2 Erciyes University Faculty of Health Sciences, Department of Nutrition and Dietetics, Kayseri, Turkey; 3 Erciyes University Faculty of Medicine, Department of Pediatrics, Unit of Social Pediatrics, Kayseri, Turkey; 4 Erciyes University Faculty of Medicine, Department of Family Medicine, Kayseri, Turkey; 5 Erciyes University Faculty of Medicine, Department of Pediatric Endocrinology, Kayseri, Turkey

**Keywords:** weight, height, percentile, children, adolescents, GAMLSS method

## Abstract

**Objective:**

To compare updated weight and height percentiles of 6-17-year-old children from all socio-economic levels in Kayseri with previous local references and other national/international data.

**Methods:**

The second study “Determination of Anthropometric Measurements of Turkish Children and Adolescents study (DAMTCA II)” was conducted in Kayseri, between October 2007 and April 2008. Weight and height measurements from 4321 (1926 boys, 2395 girls) school children aged between 6 to 17 years were included in this cross-sectional study. Using these data, weight and height percentile curves were produced with generalized additive models for location, scale and shape (GAMLSS) and compared with the most recent references.

**Results:**

Smoothed percentile curves including the 3^rd^, 5^th^, 10^th^, 15^th^, 25^th^, 50^th^, 75^th^, 85^th^, 90^th^, 95^th^, and 97^th^ percentiles were obtained for boys and girls. These results were compared with DAMTCA I study and with two national (İstanbul and Ankara) and international data from Asia and from Europe.

**Conclusion:**

This study provides updated weight and height references for Turkish school children aged between 6 and 17 years residing in Kayseri.

**WHAT IS ALREADY KNOWN ON THIS TOPIC?**

Among a number of indices for which reference standards are available; height and weight are the most useful in pediatric daily practice. Height and weight are the most easily obtained anthropometric indices. These indices have been used extensively in screening and monitoring of growth and have the advantages of simplicity and low cost for use in large-scale epidemiologic studies. National height and weight reference curves produced for Turkish children and adolescents living in Ankara (the capital city in Central Anatolia region), and İstanbul (the biggest and the crowded city in Marmara region), other than our data, are not comprehensive and has relatively big data set.

**WHAT THIS STUDY ADDS?**

The aims of the current study were; to present the updated reference data on height and weight for Turkish children and adolescents aged 6-17 years living in Kayseri, Turkey produced with generalized additive models for location, scale and shape (GAMLSS) and to compare these updated data with our previous study conducted three years earlier along with other recent references.

## INTRODUCTION

Growth and development of children are sensitive indicators of the general health and nutritional status of a population (1). The scarce studies on growth conducted in Turkey were based on children from large cities, representing relatively high socio-economic classes (2,3,4,5,6,7).

The concern about the worldwide increase in the prevalence of overweight and obesity in children is well known. The prevalence has increased substantially in children and adolescents in developed countries, and 23.8% (22.9-24.7%) of boys and 22.6% (21.7-23.6%) of girls were reported to be overweight or obese in 2013. The prevalence of overweight and obesity has also increased in children and adolescents in developing countries in recent years, from 8.1% (7.7-8.6%) to 12.9% (12.3-13.5%) in boys and from 8.4% (8.1-8.8%) to 13.4% (13.0-13.9%) in girls (8).

Turkey is a country with significant regional differences in socio-economic, demographic, and epidemiological features. This is partly due to its geography and also to past economic crises which have led to massive migratory movements of the population from rural to urban areas such as İstanbul (the biggest and most crowded metropolis) and Ankara (the capital city). Kayseri is one of the crowded cities located in Central Anatolia region of Turkey, where people used to migrate from eastern parts of the country, primarily for employment opportunities in industrial work areas.

Among a number of indices for which reference standards are available, height and weight, the most easily obtained anthropometric measurements, are also the most useful in pediatric daily practice. Height and weight are being used extensively in screening and monitoring growth. These measurements have the advantages of simplicity and low cost for use in large-scale epidemiologic studies (9).

In Turkey, national height and weight reference curves produced for Turkish children and adolescents living in Ankara (the capital city in Central Anatolia region) (10) and İstanbul (the biggest and the most crowded city in Marmara region) (6) have been reported. National height and weight reference curves have also been reported for Indian (11), Italian (12), Malaysian (13), and Polish children (14).

In our previous study [Determination of Anthropometric Measurements of Turkish Children and Adolescents I (DAMTCA I)], we reported height and weight reference values in Kayseri children and adolescents aged 6-18 years (15). After three years, during DAMTCA II study, we had the opportunity of obtaining weight and height measurements in children of the same region and to compare these two sets of data. The aims of the current study were to present the updated reference data on height and weight for Turkish children and adolescents aged 6-17 years living in Kayseri, Turkey, to produce generalized additive models for location, scale and shape (GAMLSS) (16,17) and also to compare these updated data with our previous study conducted three years earlier as well as with other recent references (6,15).

## METHODS

Data used in this study were obtained from the DAMTCA II, a cross-sectional study performed in the period between October 2007 and April 2008 for children aged between 6 and 17 years. This study was conducted in Kayseri, which is a Central Anatolian province with a population more than 1.2 million (18).

Multi-stage probability sampling was applied as the sampling method. Of the 708 schools in Kayseri, 17 (primary and secondary schools) were selected to randomly recruit children and adolescents aged between 6 and 17 years. Chronological age was calculated as the decimal age by subtracting the observation date from the birth date. The study protocol was approved by the Ethics Committee of Erciyes University and by the local educational authority. Children with any disorder affecting growth such as a known systemic or local disorder, metabolic, gastrointestinal or neurological condition, and using of any kind of medication were excluded. Parental written consent was obtained prior to the study, and the procedures were in accordance with those outlined in the Declaration of Helsinki (18).

Body weight was measured by bioelectrical impedance analysis (BIA), with Tanita BC-418 MA (Tanita Corporation, Tokyo, Japan) with correction for light indoor clothing. Height was measured with a portable stadiometer (SECA, Germany) sensitive to changes up to 1 cm. Daily calibration was performed to the portable devices. Height measurements were performed with the subject barefoot, the heels, hip and shoulders touching the stadiometer, and the head in neutral position with eyes gazing forward. The measurements were repeated twice, asynchronously, and the arithmetic mean was recorded for evaluation. All inter-observer correlation coefficients were calculated as 0.98.

### Statistical Modeling

Age-related height and age-related weight z-score plots were checked and the discontinuities were examined to filter outliers. Liberal cut-off values were used as criteria to identify outliers (19). After filtering detected outliers, the remaining 4321 observations (1926 boys, 2395 girls) were split into training (70%) and test (30%) sets randomly. The training set was used to build models and the test set to select and validate models. GAMLSS were used to build the models, for each gender separately (20). For each gender and each measurement, LMS, LMST, and LMSP methods were applied to data. Box-Cox normal (BCN), Box-Cox t (BCT), and Box-Cox power exponential (BCPE) distributions were applied for these methods, respectively. To estimate the distribution parameters, maximum penalized likelihood method was used with RS algorithm and Fisher scoring method. Cubic splines were used as smoothing functions. Analyses were applied using GAMLSS package (version 4.3-1) of R 3.1.1 software (www.r-project.org).

### Model Building

In order to apply the LMSP method, we followed the optimization procedure of Rigby and Stasinopoulos (21). Here, Akaike’s information criteria are used to select best models with parameter #3. At first, identity function was used as link functions for parameters that may relate to μ (median) and ν (skewness parameter), log-link function was used as link functions for σ (coefficient of variation) and τ (kurtosis parameter) (21). A grid search is applied for λ (power) between -2 to 2 in steps of 0.25, and an initial age transformation was optimized as x=age^λ^. Next, initial degrees of freedom (df) of all four distribution parameters was taken as 1 and df (μ), λ and df (σ) values were optimized respectively. A grid search (between 1 to 20 in steps of 1 for df (μ) and df (σ); between -2 to 2 in steps of 0.05 for λ) were applied to optimize these parameters. Next, df (ν) and df (τ) parameters were optimized with a search ranging between 0 to 9 in steps of 1. Finally, fine tuning was conducted for the model with optimum parameters with changing values of df (σ), df (μ), df (ν), df (τ), and λ. Generalized Akaike Information Criteria (GAIC) was used for model comparisons. We followed the same procedure for LMST and LMS methods, considering the absence of kurtosis parameter τ in BCN distribution of LMS method. For each gender, each anthropometric measure, and each method, final models are given in Table 1.

A two-sided independent samples t test was applied for between gender comparisons (Table 2).

## RESULTS

Results given in Table 1 reveal that LMS was detected as the best method to fit age-related height in both genders and weight in boys. LMSP was detected as the best method to fit age-related weight in girls.

Table 2 shows mean ± standard deviation values for height (cm) and weight (kg) in boys and girls. Tables 3 and 4 show age-related percentiles of 6-17-year-old Turkish boys and girls for height (cm) and weight (kg), respectively.

Tables 5 and 6 compare the 50^th^ percentile values of the current data for height and weight with other national and international studies according to gender.

Percentile curves of age-related weight measures of 6-17-year-old boys and girls are shown in Figures 1A and 1B, respectively. Percentile curves of age-related height measures of 6-17-year-old boys and girls are shown in Figures 2A and 2B, respectively.

Comparison of 3^rd^, 50^th^, and 97^th^ percentiles of age-related weights in boys (Figure 3A) and girls (Figure 3B) among DAMTCA I, DAMTCA II, and İstanbul studies are shown in Figures 3A, 3B. Comparison of 3^rd^, 50^th^, and 97^th^ percentiles of age-related heights in boys (Figure 4A) and girls (Figure 4B) among DAMTCA I, DAMTCA II, and İstanbul studies are shown in Figures 4A, 4B.

Figures 5A and 5B show differences (% values) in height (cm) and weight (kg) between DAMTCA I and DAMTCA II for 3^rd^, 50^th^, and 97^th^ percentiles in boys and girls, respectively.

## DISCUSSION

In this study, we present cross-sectional reference percentiles and curves of weight and height in Turkish children and adolescents living in Kayseri, Turkey, produced with GAMLSS method. Cross-sectional studies can provide a record of the nutritional status for a precise period and for a specific population.

Anthropometric indices, such as body weight and height, are the simplest, easiest to obtain, non-invasive, cheapest, and most widely accepted criteria for the evaluation of growth and body composition of children and adolescents. These indices also reflect, and thus are useful in the evaluation of nutritional status and health of both children and adolescents.

The reference percentile curves for weight and height of Turkish children have first been established by Neyzi et al (7) in 1970s and have been used since. The same authors have re-published the reference curves for Turkish children aged between 6 and 18 years of age by updating the curves (6). Both studies have enrolled school-aged boys and girls representing high socio-economic level and residing in İstanbul (Marmara region of Turkey) and the authors have presented their data as “predictive” reference values.

However, there are some points that can be explained by differences in methodology of the studies in the two regions. While the İstanbul study has been conducted via longitudinal follow-up of the same children, the current study has been conducted cross-sectionally in a mixed group. Also, age of onset of puberty in children in the Istanbul group appears to reflect an earlier onset due to a social improvement process in the past few decades. In addition, different nutritional habits and limited physical activity could affect the findings in pubertal children in the Istanbul group. Reflecting on our findings, it can be said that the findings of the children in the Kayseri group reflect the socio-economic improvement process in their pre-pubertal ages but not yet in their pubertal period. We can speculate that future studies on Kayseri children will reveal the expected reflection of socio-economic improvement on onset of puberty and on growth in puberty.

It is well known that height and weight differences in children can also be due to ethnic origin and geographic settlement. The differences between our values and the centers for disease control (22) or Iran data can be explained by both ethnic and geographical differences (23). With regard to differences from İstanbul data, we underline the impact of socio-economic factors to significantly influence growth, although the same ethnic origin is shared.

Our analysis indicates that DAMTCA-II height values are somewhat lower than those of the DAMTCA-I study, on an average of 2-3 cm between the ages of 6-14 years in both boys and girls. DAMTCA-II height values in other age groups and weight values in all age groups were similar to the DAMTCA-I study results for both girls and boys. DAMTCA-II height-for-age values in the boys were lower than the Ankara sample, but similar to the height-for-age values reported for İstanbul children. Weight measurements seemed to be similar in the DAMTCA-II and Istanbul studies for boys younger than 10 years old, but similar between boys older than 10 years. DAMTCA-II and Ankara studies revealed similar results in all age groups. When compared with international references, DAMTCA-II height and weight values for both boys and girls seemed to be higher than Indian and Malaysian children, but lower than Italian and Polish children.

In conclusion, we believe that the percentile values established in this group of boys and girls of 6-to-18 age group from Kayseri are representative and can be used in the monitoring of growth of children from all socio-economic levels residing in the region.

## Figures and Tables

**Table 1:**
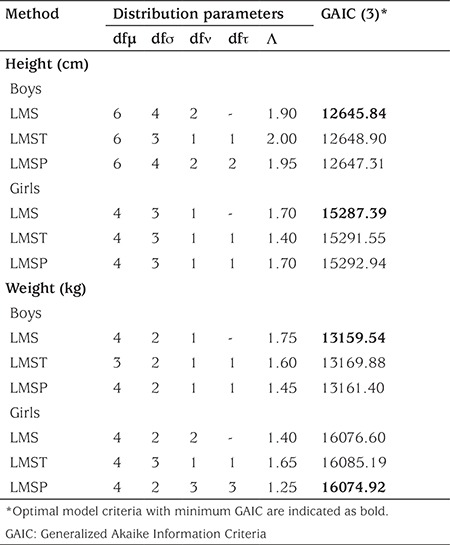
Comparison of LMS, LMST, and LMSP methods in modeling age-related height and weights for each gender in 6-17 years old children

**Table 2:**
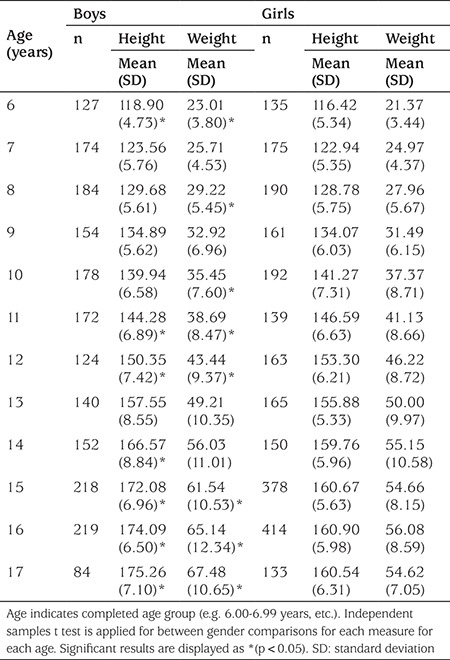
Height (cm) and weight (kg) of the study sample

**Table 3:**
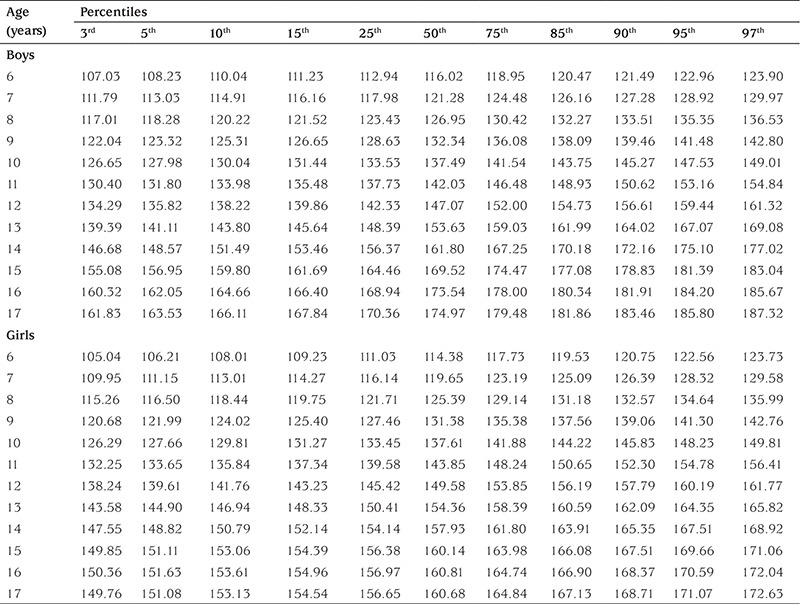
Age-related height (cm) percentiles of the subjects

**Table 4:**
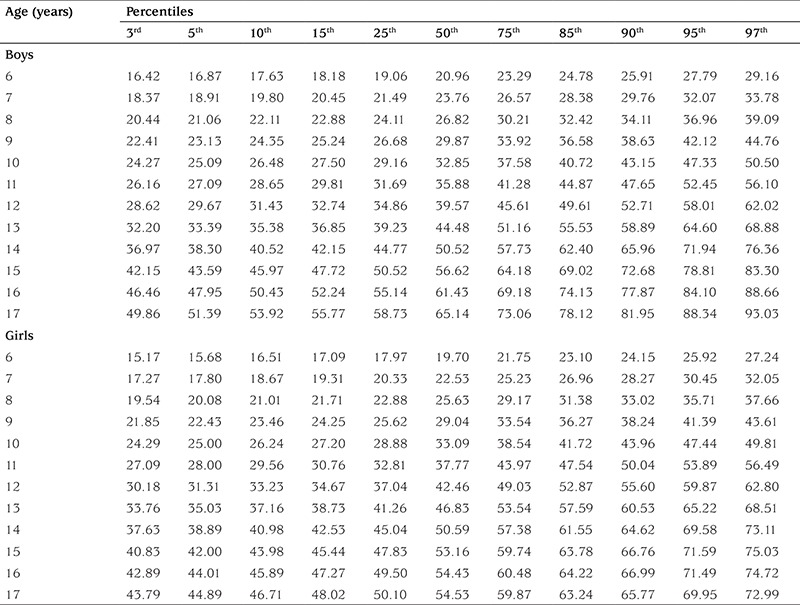
Age-related weight (kg) percentiles of the subjects

**Table 5:**
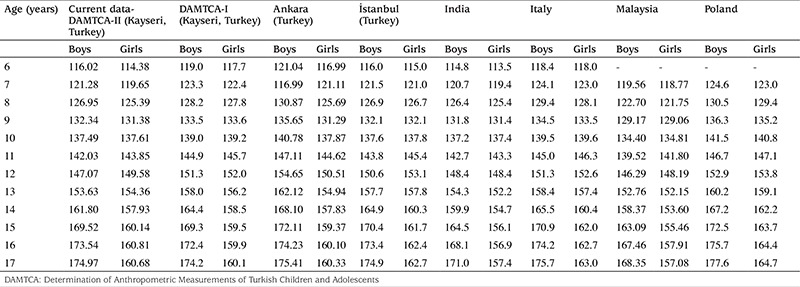
Comparison of the 50th percentile of the current data with other national and international studies (height-cm) according to gender

**Table 6:**
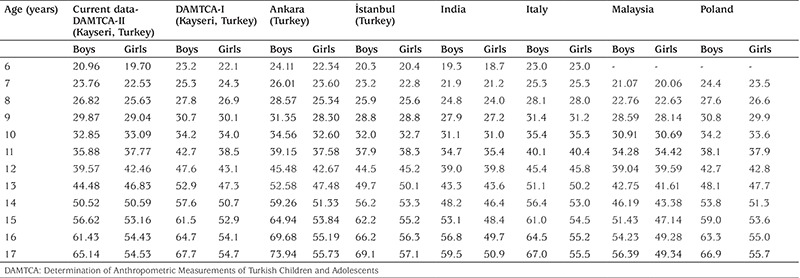
Comparison of the 50th percentile of the current data with other national and international studies (weight-kg) according to gender

**Figure 1A:**
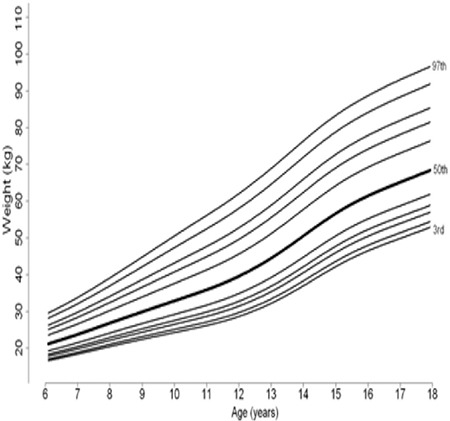
Percentile curves of age-related weight measures of 6-17 year Turkish boys

**Figure 1B:**
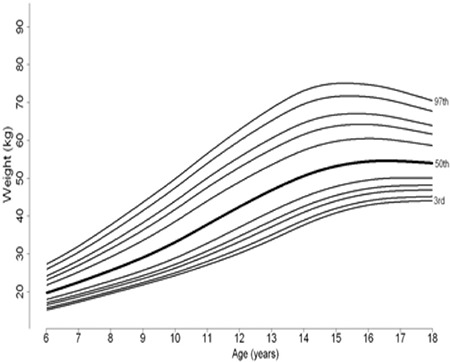
Percentile curves of age-related weight measures of 6-17 year Turkish girls

**Figure 2A:**
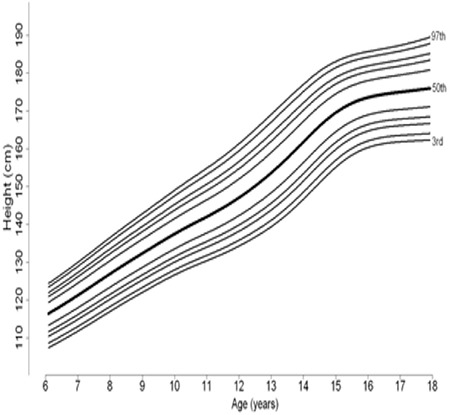
Percentile curves of age-related height measures of 6-17 year Turkish boys

**Figure 2B:**
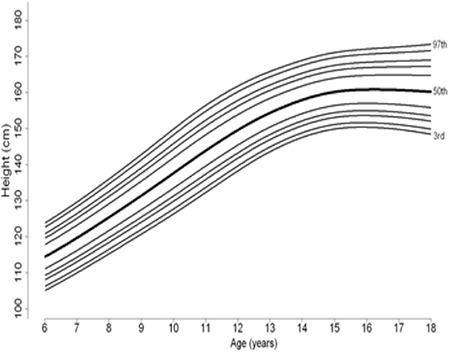
Percentile curves of age-related height measures of 6-17 year Turkish girls

**Figure 3A:**
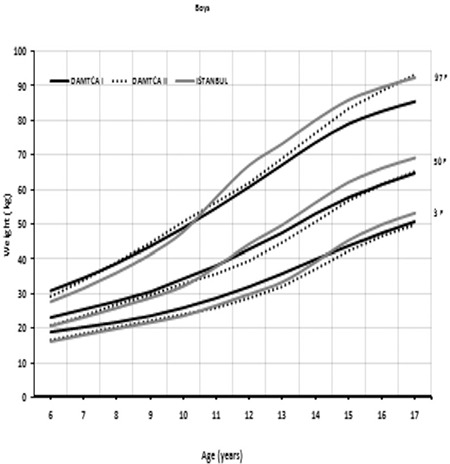
Comparison of 3^rd^, 50^th^ and 97^th^ percentiles of age-related weights in boys among DAMTCA I, DAMTCA II and İstanbul studies

**Figure 3B:**
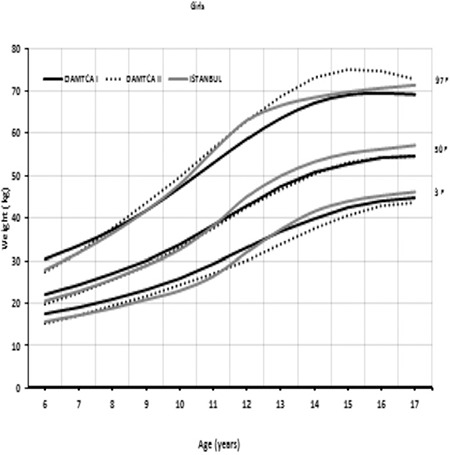
Comparison of 3^rd^, 50^th^ and 97^th^ percentiles of age-related weights in girls among DAMTCA I, DAMTCA II and İstanbul studies

**Figure 4A:**
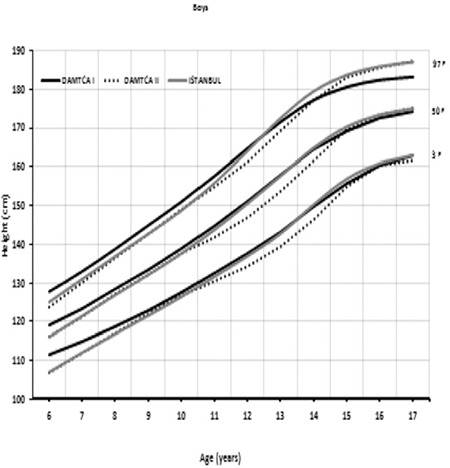
Comparison of 3^rd^, 50^th^ and 97^th^ percentiles of age-related heights in boys among DAMTCA I and DAMTCA II and İstanbul studies

**Figure 4B:**
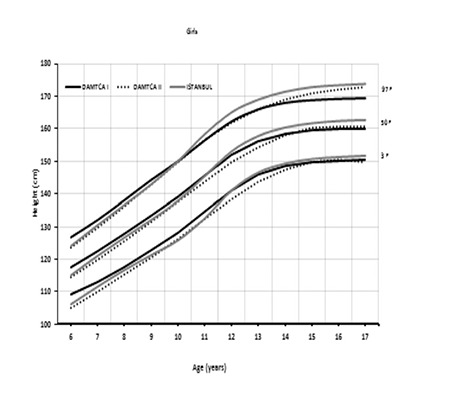
Comparison of 3^rd^, 50^th^ and 97^th^ percentiles of age-related heights in girls among DAMTCA I, DAMTCA II and İstanbul studies

**Figure 5A:**
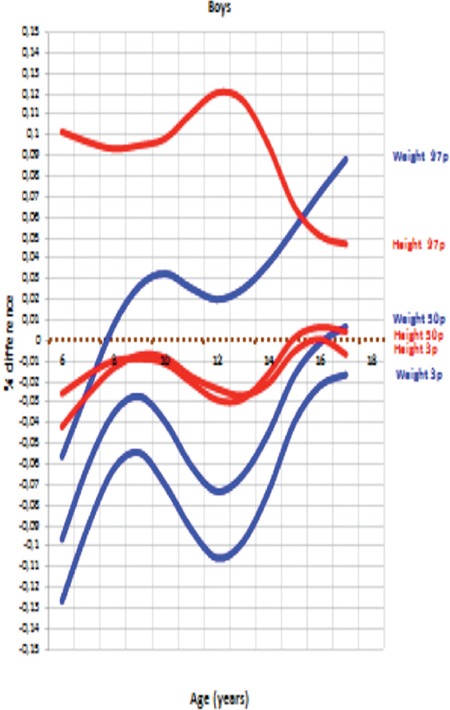
Percent (%) differences of height (cm) and weight (kg) between DAMTCA I and DAMTCA II for 3^rd^, 50^th^ and 97^th^ percentiles in boys

**Figure 5B:**
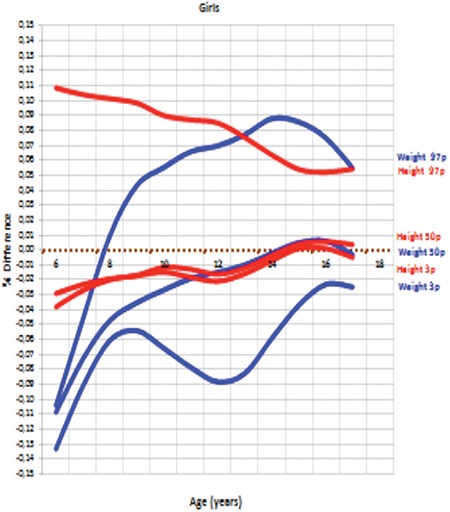
Percent (%) differences of height (cm) and weight (kg) between DAMTCA I and DAMTCA II for 3^rd^, 50^th^ and 97^th^ percentiles in girls
